# Hepatocellular carcinoma on cirrhosis complicated with tumoral thrombi extended to the right atrium: results in three cases treated with major hepatectomy and thrombectomy under hypothermic cardiocirculatory arrest and literature review

**DOI:** 10.1186/s12957-016-0831-7

**Published:** 2016-03-12

**Authors:** Benedetta Pesi, Francesco Giudici, Luca Moraldi, Gianfranco Montesi, Stefano Romagnoli, Fulvio Pinelli, Pierluigi Stefano, Giacomo Batignani

**Affiliations:** Unit of Surgery, Department of Surgery and Translational Medicine, Careggi University Hospital, Florence, Italy; Department of heart and vessels, Careggi University Hospital, Florence, Italy; Department of anesthesia and critical care-Department of healt science, Careggi University Hospital, Florence, Italy; Unit of Surgery, Department of Surgery and Translational Medicine, Careggi University Hospital, Largo Brambilla 3, 50134 Florence, Italy

**Keywords:** Hepatocellular carcinoma, Right atrium, Thrombectomy, Hypothermic cardiocirculatory arrest

## Abstract

**Background:**

Hepatocellular carcinoma (HCC) with the presence of tumor thrombus in hepatic veins and vena cava, until the atrium (RATT), is correlated with poor prognosis and with risk of tricuspid valve occlusion, congestive heart failure, and pulmonary embolism.

**Methods:**

Three patients with HCC on cirrhotic liver with RATT were studied. Operative technique, pre-operative and post-operative liver function tests, blood loss and transfusions, post-operative morbidity and mortality, and the overall survival and the disease free survival were analyzed.

**Results:**

Mean operative time was 336 ± 66 min. Intra-operative blood loss was 926.6 ± 325.9 ml. No major complications occurred. The times of hospital stay were 10, 21, and 19 days, respectively. The survival times were 90, 161, and 40 days, and the disease-free survival times were 30, 141, and 30 days, respectively.

**Conclusions:**

The complete removal of HCC with RATT may be achieved with cardiopulmonary by-pass (CPB) and total hepatic vascular exclusion (THVE). Adding the hypothermic cardiocirculatory arrest (HCCA) to the use of CPB allowed us to have minimal blood loss and hemostasis of the resectional plane. So the use of CPB and HCCA should be considered a good therapeutic alternative to the normothermic CPB with THVE.

## Background

Hepatocellular carcinoma (HCC) is the third most common cause of cancer death worldwide [1]. HCC is a peculiar tumor that has a tendency to infiltrate the vascular structures, and this characteristic, when present, has been reported as one of the most important prognostic factors for HCC [2]. Vascular infiltration occurs especially in the veins like the portal branches and more rarely the hepatic veins. This process may be complicated with the development of macroscopic tumoral thrombi with possible extension to either the portal vein trunk or the inferior vena cava/right atrium. Liver transplantation, liver resection, radiofrequency ablation (RFA), and transcatheter arterial chemoembolization (TACE) are all possible modalities to treat uncomplicated HCC, but three of them such as liver transplantation is contraindicated in the presence of macroscopic vein tumoral thrombosis while TACE and RFA have no effects on the thrombus; thus, liver resection with thrombectomy remains the only possibility to cure these patients even if there is still much of debate because of the high rate of hepatic and systemic recurrence [3].

It is well known that the presence of tumor thrombus in the hepatic veins and vena cava, with an extension up to the atrium (RATT), is correlated with poor prognosis, and the survival of patients left untreated may range from only 3 days to 2 months [4] due to tricuspid valve occlusion (ball valve syndrome), congestive heart failure, or pulmonary embolism. Liver resection with cavo-atrial thrombectomy seem to remain, however, in this urgent situation, the only effective therapeutic option with reported survival time ranging from 5 to 56 months [5].

While the complete removal of a liver tumor along with its neoplastic thrombus reaching the vena cava may be achieved with a total vascular exclusion of the liver, when it further grows and reaches the right cardiac chambers, the use of a cardiopulmonary bypass (CPB) became advisable [6, 7] even if not always mandatory [8]. Each of these reported techniques has pros and cons that may be summarized as increased blood loss, incomplete thrombus removal, and ischemic organ damage. Adding the hypothermic cardiocirculatory arrest (HCCA) to the use of CPB has been reported as safe for several retroperitoneal malignancies with the advantages of a reduced blood loss and better and thorough thrombus removal with lesser ischemic damage [9]. Major concerns and drawbacks of this technique however are represented by the presence of a preoperative liver impairment and by the duration and the deepness of the HCCA. In 2000, Wu et al. have reported the first case of hepatectomy with RATT removal using HCCA in a cirrhotic liver [10], and since then, we have not assisted to other reports, except our first patient in 2010, that showed very good postoperative outcome [11].

The aim of this study is to report the intra- and post-operative results and outcomes of three cases of HCC with RATT surgically treated with the use of CPB and HCCA, which represents at present, to the best of our knowledge, the largest series worldwide.

## Methods

All patients underwent preoperatively to a selective transcatheter arterial chemoembolization (TACE) of the main mass and then a week later by a left and a right portal embolization performed respectively in the second and third patient to stimulate the growth of the future remnant liver. Instead, in the first patient, given the presence of a complete thrombosis of the left portal vein, the portal embolization was not performed.

All patients were staged as child A according to the Child-Pugh classification and liver function evaluated with conventional liver function tests and with indocyanine green hepatic clearance test (ICG test) in order to assess the quantity of parenchyma to be removed according to Makuuchi flow chart [12].

All patients had a chest X-ray, ultrasonography (US) of the abdomen, and contrast computed tomography (CT) or magnetic resonance imaging (MRI) of the chest and abdomen.

### Anesthesia

All the described patients received the same anesthesia standardized as follows. Atropine 0.5 mg IM and diazepam 5 mg per OS were administered as premedication 30 min before surgery. After monitoring (electrocardiogram, invasive arterial pressure, SpO_2_), the induction of the anesthesia was achieved with sufentanil 0.5–1 mcg/kg and midazolam 0.1 mg/kg and maintained with propofol 2.5–4 mg/kg/h and remifentanil 0.2–0.4 mcg/kg/min at the discretion of the attending anesthesiologist. Muscle relaxation was achieved with rocuronium 0.6 mg/kg before tracheal intubation, while during surgery, it was administered by continuous infusion at the dose of 0.3 mcg/kg/min. The lungs were ventilated with 50 % oxygen in air using a semi-open circle system. Tidal volume was set at 8 ml/kg (ideal body weight), and ventilatory rate was adjusted to keep the arterial carbon dioxide partial pressure between 35 and 40 mm Hg. A positive-end expiratory pressure (PEEP) of 5 cmH_2_O was applied until CPB started and ventilation interrupted. After anesthesia induction, a three-lumen central venous catheter was placed into the internal jugular vein (echo-guided procedure) for drug administration and central venous pressure monitoring. Body temperature was measured with rectal and nasopharyngeal probes. Before the initiation of CPB, the patients received porcine heparin at an initial dose of 300 U/kg, injected IV before cannulation of the aorta. An additional dose of 5000 U of heparin was administered when the kaolin activated clotting time (ACT) was <400 s, while an additional dose of 2500 U was given if the ACT declined below 300 s. CPB pump flow was maintained at 2.4 l/min/m^2^, and mean arterial pressure (MAP) target was 70 mmHg. Norepinephrine was administered at continuous infusion to increase MAP up to the target. Hemodynamic monitoring was arterial pressure and transesophageal echocardiography (TEE)-based until CPB starts. After CPB initiation, the ventilator was stopped and the body temperature was lowered, by means of the heat exchanger, up to 24, 25, and 28 °C in the first, second, and third patient, respectively. After the target temperature was reached, 30 mg/kg of methylprednisolone, 250 mg of thyopentone, and 250 ml of 18 % mannitol solution were administered, and 10 min later, the pump flow was stopped for the surgical procedure (hypothermic circulatory arrest). At the end of the surgical procedure, the patients were re-warmed to 37 °C, resuming a spontaneous heartbeat without the need of defibrillation. The TEE was used as the weaning phase of the patient from the artificial circulation. After complete weaning from CPB, heparin was neutralized by IV infusion of protamine hydrochloride at the dose of 0.6–1 mg per 100 U of heparin administered. Heparin neutralization was considered adequate if post-protamine ACT value was within 10 % of the pre-heparin value. All patients received fresh frozen plasma at the dose of 10–15 ml/Kg. The presented cases preceded the acquisition of a rotational thromboelastometry (ROTEM) device, now used for *point-of-care* coagulation monitoring and management. The hematocrit target was 25 % but no patients received packed red blood cells during surgery. After the procedure, all the patients were admitted in intensive care unit (ICU) under sedation with propofol (1 mg/kg/h) and remifentanil (0.1 mcg/kg/min) until complete re-warming, hemodynamic stabilization, and exclusion of abnormal bleeding. All the patients were extubated within 5 h from the end of the intervention. One patient received four units of red blood cells for excessive post-operative bleeding without requiring surgical re-exploration.

### Surgical procedure

At operation, the patient was placed in supine position and the abdomen explored through a bilateral sub-costal incision with xiphoid extension. I.O. ultrasound was accomplished in all cases to rule out the presence of contralateral liver tumors or metastases and to better define the liver anatomy and the relationships between the tumor and the vascular-biliary structures. Liver resection was carried out first, tying and cutting the arterial and portal branch, and the bile duct of the hemi-liver was removed and then parenchymal transection was performed using a Kelly crush technique under intermittent Pringle maneuver lasting 10 min with 5 min of de-clamping. In the patient who had a complete thrombosis of the left portal vein with an extension into the right branch, a thrombectomy was performed, clamping the main trunk of the portal vein, opening the confluence, and extracting it from the right branch, using forceps and a Fogarty catheter. Liver resection was extended to the anterior wall of the vena cava, leaving the liver attached at the hepatic vein containing the tumor thrombus.

The xiphoid incision was extended up to the jugulum and a median sternotomy performed. The pericardial sac was opened and, after systemic heparinization, the cannulation of the ascending aorta and of the right atrium with a “vent” in the right superior pulmonary vein was accomplished in order to perform an extracorporeal circulation with CPB. Then, the CPB was started and the body temperature lowered by systemic cooling and saline solution iced “slashed” that was placed in the pericardium and around the remnant liver in order to protect more these organs. There was no additional brain protection during HCCA. After HCCA had started, the right atrium and the vena cava were opened longitudinally and the thrombus removed. After the closure of the atrium and vena cava by means of 4/0 prolene continuous running suture, the patient was re-warmed and weaned from the CPB. HCCA median time was 14 min (range 12–24).

**Fig. 1:**
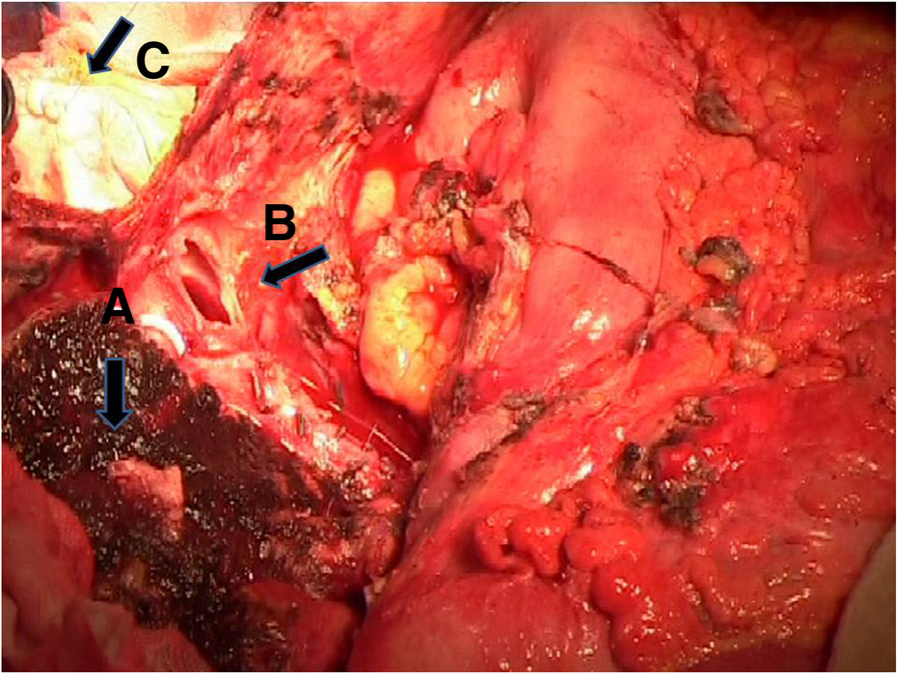
Intraoperative view of the resected liver during hypothermic cardiocirculatory arrest. Please note the complete bloodless field with the transected middle hepatic vein (*A*, *B*) after the tumoral thrombus removal from the atriotomy site (*C*) already sutured

In the two cases of HCC of the left hemi-liver in whom the thrombus intersected the confluence of the left and middle hepatic veins (HVs), two different ways were undertaken. In one case, the middle HV was preserved by suturing the outlet of the left HV in the middle HV, once the thrombus had been removed. In the other case, the confluence was resected (Fig. 1) and during the re-warming phase, we reconstructed the continuity of the middle hepatic vein by means of an interposition of PTFE ringed graft of 8-mm diameter anastomosed to the inferior vena cava.

Pathology of the specimen showed a multifocal moderately differentiated hepatocellular carcinoma and focal clear cell with vascular invasion on cirrhosis.

## Results

Three patients, two males and one female with ages 51, 73, and 76 years, had a primary liver tumor (HCC) with a thrombus extending to the supra-hepatic vena cava and to the right atrium. The tumor diameters were 6.5, 4.5, and 14 cm, respectively. Two patients had a tumor in the left hemi-liver, with a thrombus invading the left-middle HV and extending through the supra-hepatic vena cava to the right atrium. The first of these two patients had also an invasion of the left portal vein with an extension into the right branch while the second had a segmental neoplastic pulmonary embolism of the left pulmonary artery. The third patient has had two tumors of the right hemi-liver with invasion of the right HV and extension to the right atrium.

The pre-operative liver function tests are shown in Table 1.

**Table 1:** Pre-operative liver function tests

	PT (%)	Albumin serum (g/dL)	Bilirubin serum (g/dL)	SGOT (U/L)	Indocyanin green retention test (%)	Alfa-fetoprotein (ng/dL)
Patient 1	76	4.1	1.8	53	22.3	8.6
Patient 2	74	3.6	1.1	60	23.5	12.4
Patient 3	83	5.1	1.09	76	19	21.5

Mean operative time was 336 ± 66 min. Intra-operative blood loss was 926.6 ± 325.9 ml. No patients required intra-operative blood transfusions and only one patient received four units of blood post-operatively for a hemoglobin (Hb) level dropped to 7.8 g/dl without signs of active bleeding and no drainage output.

**Fig. 2:**
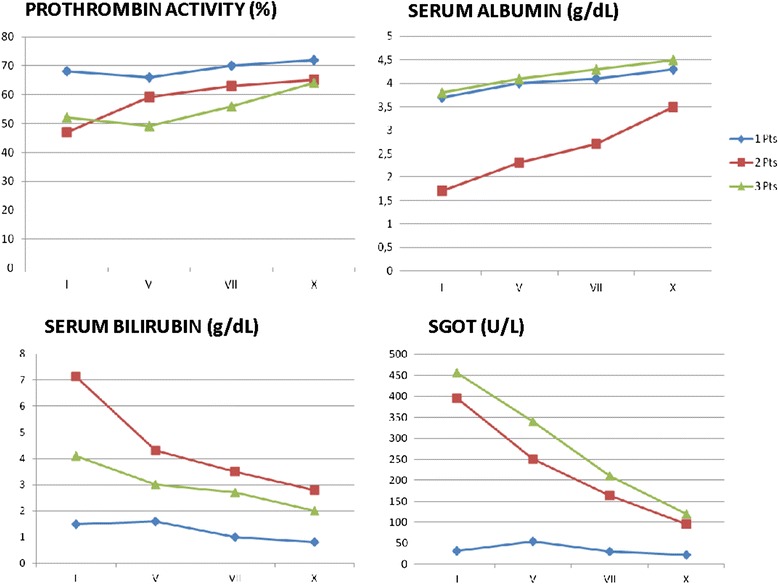
Postoperative liver function tests

All liver function tests returned to normal within 1 month of the operation. The postoperative liver function tests are shown in Fig. 2. The patients had uneventful recovery except for one patient who had right pleural effusion and abdominal ascites that had to be drained and treated with medical therapy as diuretics and albumin. The times of hospital stay were 10, 21, and 19 days, respectively. The survival times were 90, 161, and 40 days, and the disease-free survival times were 30, 141, and 30 days, respectively.

## Discussion

HCC tends to invade the intra-hepatic vasculature, especially the portal vein [13]. The tumor thrombosis of the hepatic veins and vena cava up to the right atrium is uncommon, with a percentage of incidence reported of 2.9 % by imaging techniques, 0.7 % at operation, and 18.2 % at autopsy in Japan [14]. The therapeutic options at this stage are few and debatable [3]; however, Lin et al. [4] reported a median survival of few days in the untreated patients. The presence of macroscopic tumoral thrombosis contraindicates liver transplantation. The role of TACE in patients with HCC and caval/atrial thrombosis has been recently investigated by Wang et al. in 2012 who reported no difference in median survival time between patients who underwent TACE and those who did not received any treatment. Furthermore, the same authors reported a great and significative advantage of a third group with caval/atrial thrombosis, surgically treated, which showed a survival of 68, 22, and 13 % at 1, 3, and 5 years even if there were only 3 of the 25 patients in whom the thrombus had reached the right atrium [6].

RATT has major complications as “ball valve syndrome” [15], acute pulmonary embolism [16], or congestive heart failure, situations that represent an impending life-threatening condition that should be surgically treated as soon as possible and being the liver resection with thrombectomy, the only therapeutic option in this setting [10].

From an oncological point of view, however, there still much of debate about the high recurrence rate and the real gain in terms of life expectancy in these surgically treated patients. Anyhow, a review of 18 cases operated in Japan resulted a mean survival of 11 months with three patients alive for more than 3 years [17]. These data were confirmed by two further studies which showed a mean survival of 11 and 19 months [6, 7] with three patients alive for more than 5 years and with the longest survivor of 105 months [6].

From a technical point of view, the RATT can be removed in three different ways. The first is to perform a total hepatic vascular exclusion (THVE) of the liver without the use of the CPB [18, 19] but this technique is indicated for thrombi initially abutting the right atrium that can be reduced manually or lowering the liver and the thrombus in the IVC clamping it thereafter above the thrombus [18] but with the risk of an incomplete resection [20]. The second possibility, more largely used and reported, is to use a normothermic CPB coupled usually with THVE [5–7, 14, 16, 17, 21–24].

The use of extracorporeal circulation with HCCA in cirrhotic patients has been limited for many years because of the fear of intra-operative bleeding, possible brain damage, and post-operative liver failure that might result in a poor outcome.

Historically in 1981, Ein et al. [25] used HCCA to remove RATT from hepatoblastoma in a young child with normal liver parenchyma. This was the first time that CPB with HCCA was used in liver surgery and was followed by Wu et al. who used it to remove an RATT along with a wedge liver resection of a recurrent HCC on a cirrhotic liver [10]. Indeed, these authors added the use of a deep HCCA (18 °C) to the CPB, as a rescue for massive bleeding due to the dense adhesions around the liver hilum, that rendered impossible to perform a THVE and to resect the liver parenchyma first, which is advisable before the systemic heparinization, and for these reasons, they complained of 7 l of blood loss. Ohwada et al. [26] used CPB with mild hypothermia at 29 °C for the successful “en bloc” resection of a HCC in a cirrhotic liver with a RATT but these authors used the THVE instead of a proper HCCA. In 2009, we described our first case of HCC on a diseased liver with tumoral thrombus extension in the hepatic vein and vena cava which reached the right atrium treated with an extended left hepatectomy and tumoral thrombus removal using a CPB with mild HCCA at 24 °C [11]. Since then, there were no other cases reported in literature of major hepatectomy with RATT removal with CPB and HCCA on a diseased liver and then we think that even if our series is small with only three patients, it could be of unique interest.

All our three patients belonged to the child class A and underwent to the assessment of the liver function by means of indocyanine green retention test to demonstrate the presence of a good functional reserve [27] but percentages less than or equal to 25 % were deemed acceptable because of the presence of the thrombosis of a one or two hepatic vein/s which had already negatively affected the liver functionality. However and moreover, we preferred to add a selective TACE of the main mass and a portal embolization, of the hemi-liver which had to be removed, except in the case where the left portal branch was already closed by a tumoral thrombus. We think in fact that adding a sequential arterial and portal embolization of a hemi-liver could lessen the possibilities of post-operative liver failure when a major resection is planned [9].

Our surgical strategy entails the liver resection first, using an intermittent Pringle maneuver, before going on CPB and HCCA according to other authors [6, 14]. This has allowed us to have minimal blood loss through a thorough hemostasis of the resectional plane before heparin administration, and surprisingly enough, we did not notice any bleeding during the re-warming phase and after the heparin neutralization. In fact, we have had in all our patients a blood loss comparable to that of major hepatectomies with only one patient who received only four units of blood postoperatively. This appeared quite different from the data reported in a review from Japan where the total blood loss ranged from 3 to 14 l [22] and later by Wakayama et al. in a single institution series where a median of 6 l with range from 1 to 35 l was complained in six patients with RATT operated using CPB with THVE [7]. We have preferred to use a mild hypothermia at 24 to 28 °C instead of a deep one, at 18 °C used by Wu et al. [10], because the time necessary to remove the atrial thrombus along with the liver was expected to be within 30 min and this may have contributed to a better maintenance of the hemostasis. Furthermore, there are increasing data supporting the less negative effects of the hypothermic versus the normothermic liver ischemia determined when THVE is used [28, 29]. We have had a median HCCA time of only 14 min during which the thrombi were thoroughly removed, in a complete bloodless field, from the right cardiac chambers and in one occasion from its propagation into a phrenic vein as well as, in another patient, to remove an embolus in the left pulmonary artery. This supports the fact that HCCA may be superior to the other techniques in order to obtain a better cleaning of the tumoral material as previously reported in kidney surgery [9].

Despite the fact that there is a lot of concern about the high operative risk, recently, Wang et al. [6] demonstrated that the liver resection with caval/atrial tumor thrombectomy can be done with little morbidity and no mortality using CPB and THVE. Accordingly, we also did not experience any postoperative death, and major postoperative complications have been negligible, except in one patient who showed a prolonged output of ascites from the drainages, medically treated with albumin and diuretics infusion, which resolved in few days, in our patients using HCCA. So we think that this surgical technique, with a short lasting mild hypothermic circulatory arrest, should be considered for the removal of atrial thrombi also in the presence of a well-compensated liver cirrhosis.

## Conclusions

In conclusion, the type of surgical approach is not still standardized in HCC with tumor thrombosis involving the hepatic and caval vein up to the heart, but we think that a combined thoracic and abdominal surgery with the use of CPB and HCCA should be considered a good therapeutic alternative to the normothermic CPB with THVE in the presence of a well-compensated liver cirrhosis. HCCA in fact seems to be superior to THVE in order to reduce the amount of blood loss and to obtain a better and thorough cleaning from the tumoral thrombus. Furthermore, the sequential arterial and portal embolization should be performed to stimulate the liver growth and to improve the liver function in order to obtain low postoperative complications.
